# Supplementary material for: Growth Performance and Fecal Microbiota of Dairy Calves Supplemented with Autochthonous Lactic Acid Bacteria as Probiotics in Mexican Western Family Dairy Farming
Source: Animals (Basel). 2023 Sep 7;13(18):2841. doi: 10.3390/ani13182841 (PMC10525134; doi:10.3390/ani13182841)
Supplement: Supplementary file 1 [file animals-13-02841-s001.zip › animals-2520151-supplementary.pdf]

Growth performance and fecal microbiota of dairy calves supplemented with autochthonous lactic acid bacteria as probiotics in Mexican western family dairy farming

Supplementary material

Table S1. Weight and height gain at eight weeks of Holstein calves treated with probiotics during the milk-feeding period.

	**Treatment**				***p*-value**
**Parameter**	**Control**	**Strain 6BZ**	**Strain 6BY**	**6BY+6BZ**	
Daily weight gain, kg/d*	0.627±0.04	0.598±0.05	0.669±0.05	0.682±0.03	0.456
Daily height gain, cm/d*	0.287±0.02	0.218±0.02	0.241±0.02	0.269±0.01	0.080

* Average value ± standard error.
